# The design of a laboratory apparatus to simulate the dust generated by longwall shield advances

**DOI:** 10.1007/s40789-019-00273-4

**Published:** 2019-10-14

**Authors:** Michael R. Shahan, William Randolph Reed

**Affiliations:** 1 CDC NIOSH, Pittsburgh, PA 15236, USA

**Keywords:** Coal mining, Longwall shields, Dust control

## Abstract

A laboratory apparatus (shield dust simulator) was designed and constructed to simulate the dust generated during the advance of longwall hydraulic roof supports, or shields. The objective of the study was to develop a tool that could be used to test the hypothesis that foam applied to a mine roof prior to a shield advance could be used to reduce the respirable dust generated during shield advances. This paper will outline the design parameters for the development of the system, as well as describe baseline testing of coal and limestone dust. Results show that the average instantaneous respirable dust concentrated during simulated shield advance. Confidence intervals were calculated from the instantaneous respirable dust data to determine the repeatability of the data produced by the device.

## 1 Introduction

During longwall mining, miners can be exposed to respirable dust that is generated during the advance of hydraulic roof supports or shields. National Institute for Occupational Safety and Health (NIOSH) researchers have conducted numerous surveys of longwall mining operations, benchmarking the operating practices and dust control measures in use. It has been determined that the shields are responsible for 27% of the respirable dust that is generated on the longwall face (Rider and Colinet 2011; Jankowski and Organiscak 1983). The dust generated by the shield advances ranks second, followed by the longwall shearer which is accountable for 43%. The respirable dust generated during the advance of the shields can be a significant contributor to the respirable dust exposure of the mining personnel operating at the longwall face, especially since some of the dust are generated in the walkway (Srikanth et al. 1995). Control systems designed to reduce the dust content levels generated during the shield advance have the potential to greatly reduce the respirable dust exposures of the personnel at downwind of mining activities (Chekan et al. 2010).

One of the control technologies to decrease the dust generated by the shield advances would be to apply a layer of foam at the forward shield-roof interface prior to the shield advance (Reed et al. 2018a). The blanketed layer of foam would interact with the material located on the top of the shield during the advance, preventing a portion of dust from becoming entrained in the mine air. This layer of foam would be applied by using a foaming nozzle located on the longwall shearer body that would spray foam on the roof surface as the shearer passed (Reed et al. 2018b). Substantial laboratory work has been completed to determine the feasibility of this type of foam application. NIOSH engineers have classified the various characteristics of foaming agents, using foam expansion ratio and drainage as important performance parameters, as well as developing the mechanical systems required to apply the foam safely to the mine roof (Reed et al. 2018a, b).

The construction of an apparatus that could simulate the mechanical interaction between the dust-laden shield top and mine roof is necessary to adequately test the hypothesis that foam could be an appropriate control. A team of engineers designed and constructed a shield dust simulator system allowing for in-house testing prior to a full-scale field investigation. And then what this paper is studying and discussing should be shortly and effectively illustrated, including former researcher’s outcome and the general idea of this article.

## 2 Test apparatus design overview

**Fig. 1:**
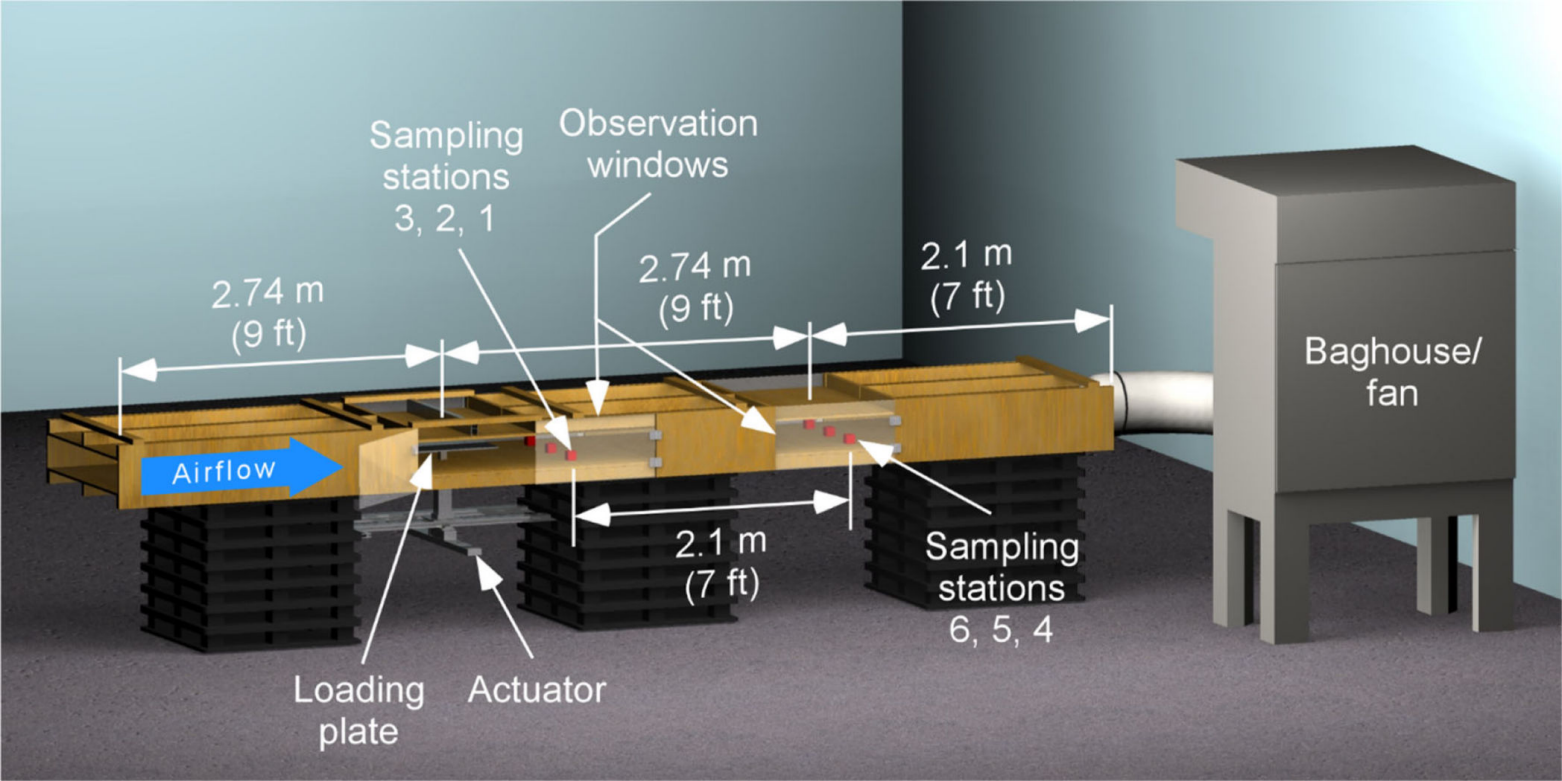
3D schematic of the shield dust simulator

A shield dust simulator was developed that could simulate the interaction between the mine roof and shield top to extract the coal dust sample of known mass that advance along a simulated mine roof and compressed. This process imitates the motion that occurs during the shield advance. Three basic processes occur during the shield advance. First, the shield depresses, releasing pressure on the shield from supporting the mine roof (lowering cycle). Next, the shield moves forward to advance toward the coal wall face (movement cycle). Lastly, the shield raises to support the mine roof, increasing pressure on the shield by the mine roof (set cycle). A system was developed that could execute these processes using pneumatic actuators. The actuators were designed to move a plate (shield) loaded with dust across a simulated mine roof by a distance of 1 m (a typical width of a longwall shearer cut), loading to the roof 0.5 m from the lowering cycle. Since the actual compressive forces produced by shields can reach over 800 metric tons (Barczak and Gearhart 1992), the actuators were not sized to produce the loading forces that are experienced during a shield move, but rather to emulate the physical process that occur during a shield operation cycle. Sampling stations were placed downwind of the test section spatially across the cross-section of the wind tunnel as shown in Fig. 1.

### 2.1 Dust wind tunnel

The test section was constructed within a wind tunnel, which allowed the air velocity to be controlled with an air regulator, simulating the face velocities found on a longwall face. Typical longwall face velocities range between 4.1 and 5.1 m/s (800 fpm and 1000 fpm) (Tomb et al. 1991). The tunnel was designed to control tunnel velocities from 3.05 m/s (low velocity, 600 fpm) to 5.1 m/s (high velocity, 1000 fpm). The wind tunnel was constructed of 12.7 mm thick plywood and 50.8 mm × 101.6 mm wood framing. All of the framing remained on the outside of the tunnel, allowing for a relatively smooth interior surface. This was designed to reduce the interference of the flow pattern within the tunnel. The cross-sectional dimensions of the tunnel were 203.2 mm × 1219.2 mm. The tunnel was 7.62 m (25 ft) in length. All of the outer seams of the tunnel were sealed with foam insulation to prevent any air infiltration that could disrupt dust entrainment. The 1219.2mm designed width housed the shield movement apparatus, allowing for the stroke of 1 m to be achieved, close to the width of a shearer drum. The 203.2 mm height was selected to simulate the distance between the highest and lowest points of shield during movement. NIOSH engineers determined that this was the area to be evaluated and it was recognized that the dust from shield drops all the way to the floor of the roadway, but the idea was to evaluate the dust concentrations as close to the source as possible and conduct evaluations of the re-entrained dust.

### 2.2 Wind tunnel fan

**Fig. 2:**
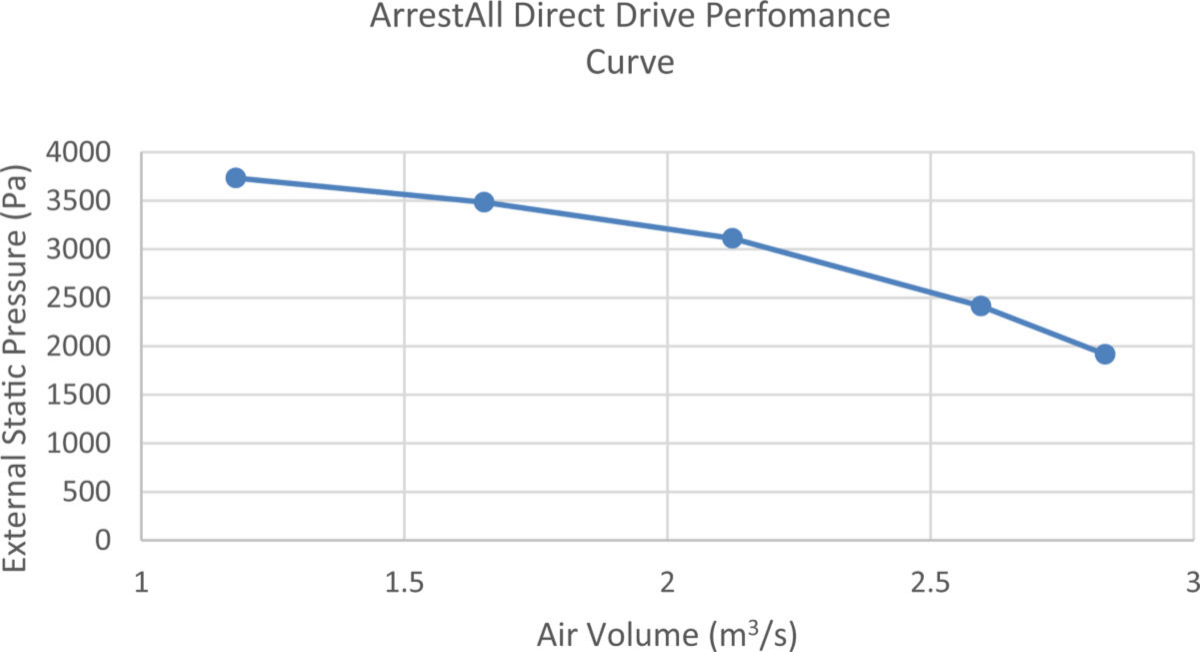
Arrestall direct drive performance curve

Wind tunnel air velocities were controlled using an Arrestall Model AR55 baghouse which has the capacity to move 1.2–2.8 m^3^/s of airflow, dependent upon fan RPM. A 61 cm diameter air damper was added to the connecting ductwork in order to allow for wind tunnel air velocity control. The fan specifications and drive performance can be found in Table 1 (Fig. 2).

The baghouse has the capability to be set at various control speeds; however, it was set to its maximum setting for the wind tunnel operation. This allowed for the two operating flow rates to be achieved using the attached control damper. Relative humidity and temperature were recorded at the tunnel’s entrance during all baseline tests for a quality control measure.

### 2.3 Test section

The test section was located 2.74 m (9 ft) from the tunnel entrance for air flow stabilization. The top surface of the test section (simulated mine roof) was designed therefore it could be removed and replaced with a fresh cleaned roof between simulations. This would allow for quick changeover of the experiment with a majority of the cleaning functions taking place outside of the test chamber. The bottom plate was also made to be removed for easy loading of the dust plate. A device was constructed with a 3D printer in aacrylonitrile butadiene styrene (ABS) to fit on the loading plate which allowed for a consistent application of dust on the loading plate surface. The dust thickness was approximately 6.35 mm and was applied to be flush with the 3D printed device. The plate was weighed to determine the amount of dust used for each experiment and these values were documented.

**Fig. 3:**
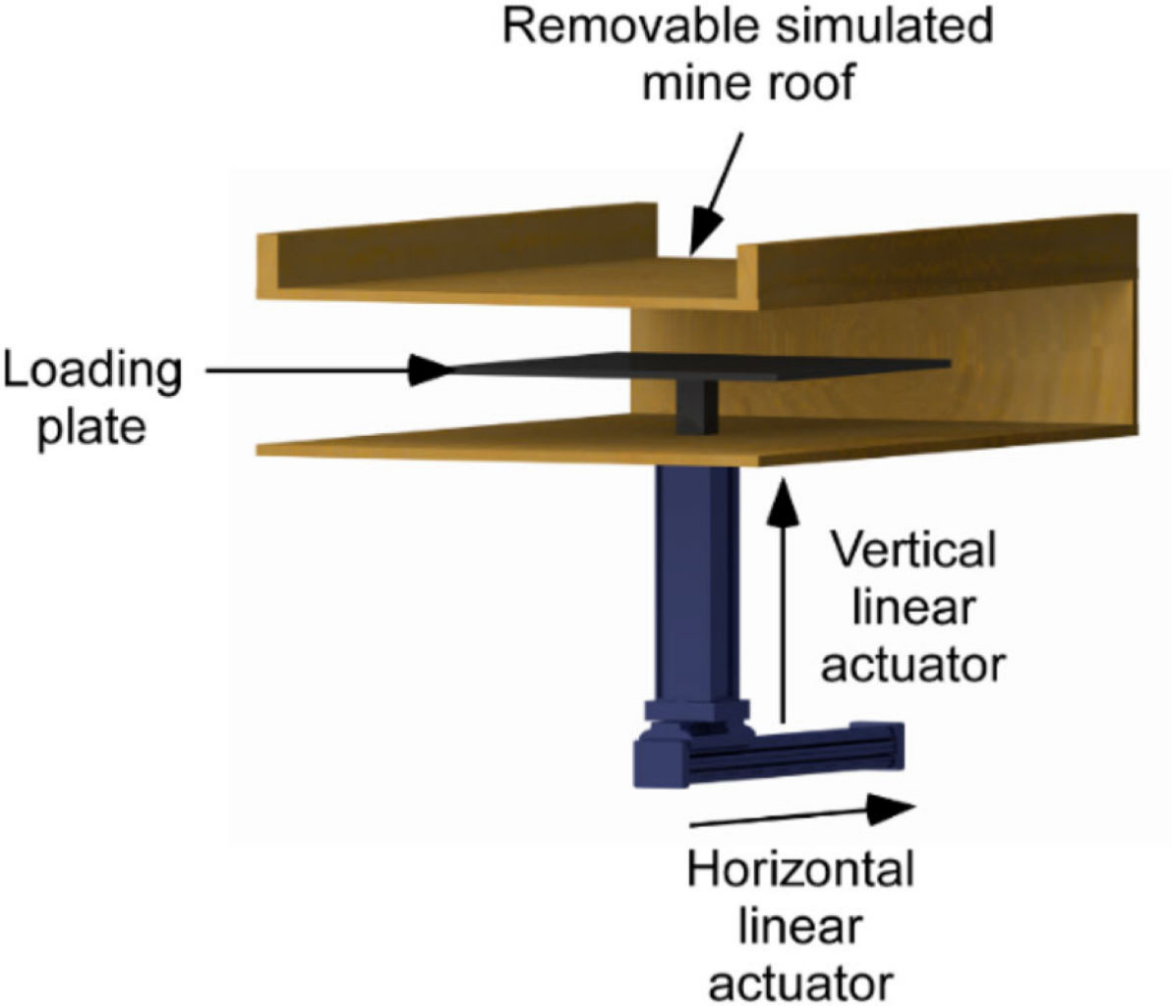
Test section 3D view

Two actuators, vertical linear and horizontal linear, were used to simulate the shield movements. A cross-sectional cutout of the test section is shown in Fig. 3.

Each pneumatic actuator was controlled using an air directional control valve with five ports and double solenoids which allowed for operation in three positions. All ports were 6.35 mm national pipe thread (NPT). A user interface was programmed using LABVIEW software to control the mechanism utilizing a data acquisition device (DAQ). This allowed for the repeatable and consistent operation of the test apparatus. A graphical user interface was developed to execute the test functions. All data was collected using the DAQ and was post-processed after the completion of the experiment. Actuator specifications can be found in Table 2.

## 3 Experimental protocol

### 3.1 Dust materials

When a shield advances, the material above the shield has the capability to be pulverized and then entrained in the mine air. Depending upon the mine, the material of the mine roof may be a combination of shales, sandstones, and coal. Using the shield dust simulator, it was determined that baseline measurements would need to be established. Keystone Black 325 BA was used for coal dust, and limestone dust was used to simulate the other non-coal material.

### 3.2 Keystone black coal dust

Keystone Black (325BA Mineral Black Filler) is used as an additive in various manufacturing processes as well as for coal dust research. Keystone Black is a refined bituminous coal dust from the Pocahontas No. 3 seam, manufactured by Keystone Filler and Manufacturing. Three samples were analyzed for particle size analysis (shown in Table 3).

### 3.3 Bagged limestone dust

The limestone is pulverized limestone dust which has < 2% silica content, 70% passing 200 mesh, and is bagged into 22.6 kg bags. The limestone dust comes from Allegheny Mineral Corp. A sample of this dust was analyzed for particle size analysis. Table 3 presents the results showing the cumulative percent passing based upon the particle size diameter.

### 3.4 Sampling methodology

Two air sampling stations were located downstream of the test section. Air sampling station No. 1 was 1 m from the test section. Air sampling station No.2 was located 2.75 m from the test section. Each station consisted of gravimetric samplers and instantaneous samplers. The gravimetric samplers were Dorr-Oliver cyclones with 37-mm, 5-μm PVC filters. These samplers were connected to a vacuum pump via a manifold that housed critical flow orifices which produced 2.0 lpm flow to each sampler. The flow rate through each orifice was verified prior to testing using a Gilbrator-2 Primary Air Flow Calibrator. Each gravimetric sampler was paired with a personal DataRAM pDR1000 Monitor (pDR). The pDR measured instantaneous dust concentration and recorded the values at a frequency of 2 Hz (or 0.5 s). The gravimetric measurement acquired during testing was used to calibrate and adjust the pDR data collected by using Eq. (1).

(1) $$pDR_{Cor.ratio}=\frac{Grav.\;filter\;conc.}{pDR_{avg.conc}}$$

(2) $$pDR_{Cor.}=pdr_{Cor.\;ratio}\times pDR_{inst.}$$

The gravimetric filter concentration is determined by the mass deposited on the filter per volume of air passed through the filter in mg/m^3^. pDR_avg.conc_ is the average concentration of all of the measurements collected by the pDR 1000 for the time of testing. The time period of the instantaneous and gravimetric samplers must be equal in order to correctly calibrate the instantaneous data. This ratio is then used to correct the instantaneous pDR_inst_ measurement by multiplying each value by pDR_cor.ratio_ (Eq. 2).

Twenty cycles of the simulated shield advance were completed for each test to ensure that sufficient mass was collected on the respirable dust filters. The filter and pDR units were located spatially 45, 76 cm and 104 cm from left sidewall of the wind tunnel (looking into the dust simulator inlet) to capture the dust gradient generated during the simulation. The sample inlet was located 10 cm from the tunnel floor. A total of 40 cycles were completed for each test condition.

### 3.5 Test procedures

A test protocol was developed to determine the baseline characteristics of the coal and limestone dust at high and low velocities. For the test, a cleaned simulated mine roof was inserted into the test chamber. The doors on the wind tunnel were closed and the tunnel fan was started. After allowing the system to stabilize for 60 s, tunnel air velocities were measured and recorded. The air velocity was maintained and adjusted to ± 0.25 m/s of the targeted velocities. Psychometric data (wet bulb temperature, dry bulb temperature, humidity, and dew point) were collected at the tunnel entrance for the duration of testing. This information was collected as a quality control measure to ensure environmental conditions were stable, not influencing changes in the test results.

Gravimetric samplers were loaded and instantaneous samplers programmed. The vacuum pump for the gravimetric was started and the pDR units began collecting data for the duration of the test.

A dust sample was loaded onto the loading plate utilizing the 3D printed ABS with 6.35 mm fixture. The sample was weighed on a microbalance and the test weight was recorded. The loading plate was then inserted onto the loading cylinder. The doors of the wind tunnel were closed and the plate was loaded to the simulated mine roof. Once loaded, the test sequence was initiated and the loading plate was cycled through the shield advance sequence.

The shield advance sequence consisted of the loading plate dropping, then moving forward, and then loading before reaching the other side of the dust chamber. The loading plate automatically returned to the home position where it could then be removed and cleaned. The shield advance sequence totaled 20 s and can be broken down into the following segments (Table 4).

This process was repeated for 20 cycles per set of respirable filters. Two sets of filters were collected for each test condition, resulting in 40 cycles per test condition. Table 5 shows each test condition that was completed.

### 3.6 Data analysis

**Fig. 4:**
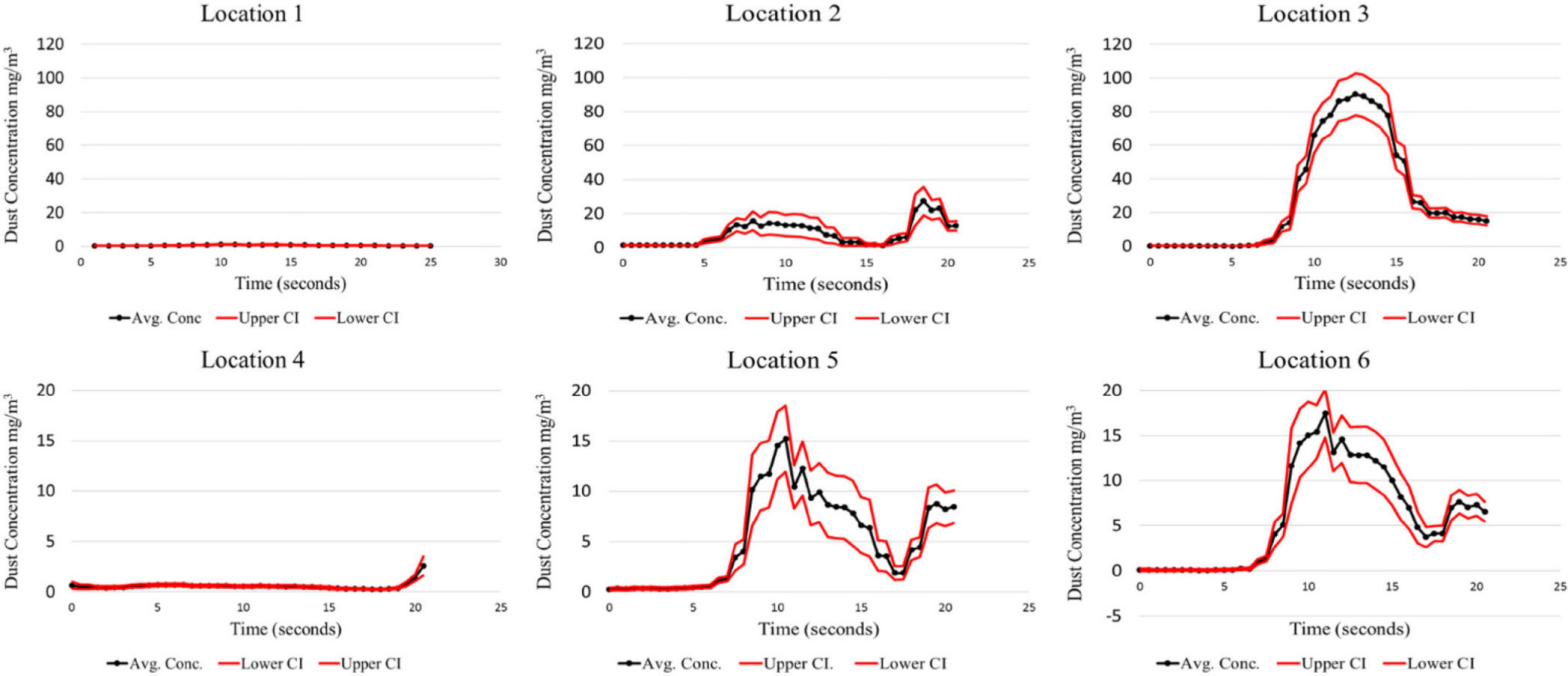
Coal dust—low air velocity baseline

**Fig. 5:**
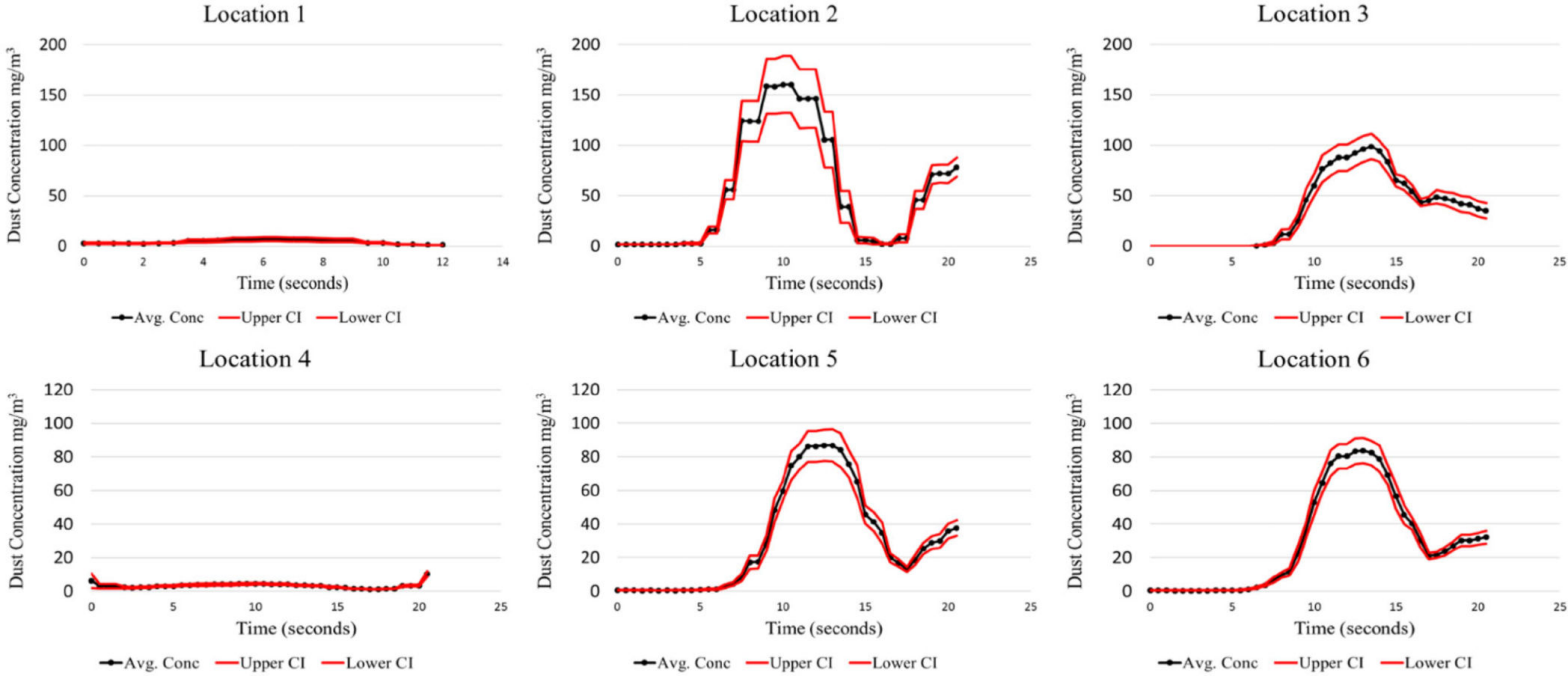
Coal dust—high air velocity baseline

**Fig. 6:**
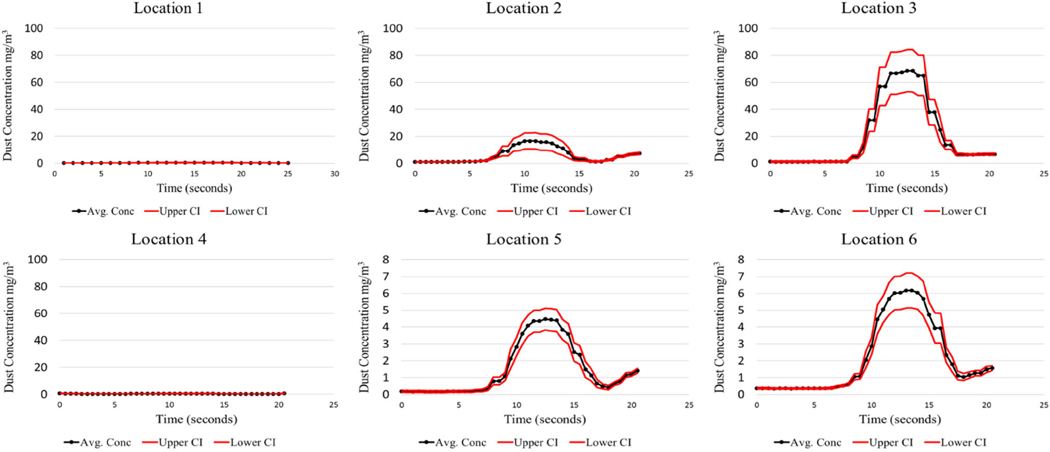
Limestone dust—low air velocity baseline

**Fig. 7:**
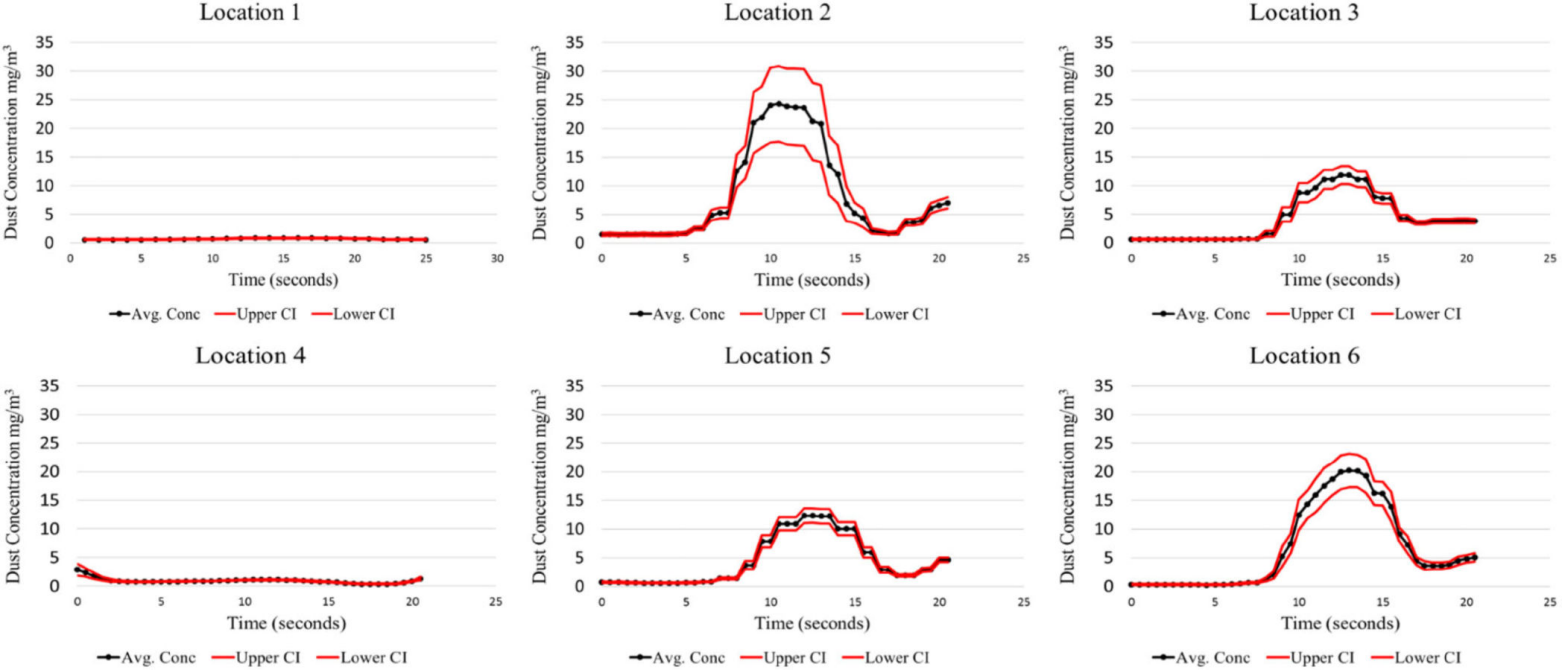
Limestone dust—high air velocity baseline

The LABVIEW program was created to initiate the test sequence, log the executed test time for each cycle, and record the instantaneous respirable dust concentrations generated during testing. This offers researchers the opportunity to analyze the dust concentrations produced during the shield movement. Since the test cycle lasted approximately 20 s, this time interval was the basis for evaluation. The analysis for each test condition evaluated all 40 cycles. The mean, standard deviation, and 95% CI were calculated for the observed respirable dust concentration over the 20-second shield advance sequence. Results from the baseline test can be found in Figs. 4, 5, 6, and 7.

It should be noted that locations 1, 2, and 3 are positioned at Station 1, and locations 4, 5, and 6 are positioned downstream at Station No. 2. There is a large concentration gradient that exists between sampling location 2 and 3. This gradient is absent at sampling locations 5 and 6. This is thought to be the case because of the air mixing taking place from the front sampling station, Station 1, to the rear station, Station 2. Interestingly, the dust concentrations as measured at Station 2, at the high-velocity test condition, peak at a much higher concentration (85 mg/m^3^) compared to the low-velocity test condition (15 mg/m^3^). The higher air velocity entrains a larger portion of the dust. Similar findings were found in Chekan et al. (2010). Higher mine ventilation rates can contribute to an increase in the amount of dust entrained in the air during shield advances.

The limestone dust behaved quite differently from coal dust, producing much lower airborne respirable dust concentrations. This was expected due to the limestone dust’s tendency to agglomerate when compressed. Sampling locations 5 and 6 have the lowest confidence intervals as seen in coal dust. This is likely attributed to the mixing that has taken place by the time the dust cloud reaches these locations, creating a more homogenous mixture.

The data collected during the baseline tests provided a visual insight and statistical analysis of the repeatability of the laboratory apparatus. This data will be used to determine the effectiveness of foam agents added to the mine roof in future studies. The data shows that the apparatus has the capability to reproduce consistent tests of dust generated during the simulated shield advance.

## 4 Conclusion

A laboratory apparatus was designed that could simulate dust that is generated during longwall shield advances. Baseline testing was conducted to characterize the dust produced during this advance, which will later be used to evaluate the effectiveness of various foam applications to the mine roof surface. From the baseline testing, it was observed that the test apparatus was capable of producing repeatable results consistently throughout all test cycles. Coal high-velocity results showed that maximum respirable dust concentrations ranged from 100 to 150 mg/m^3^ at 1 m from the source, while the maximum concentrations ranged from 80 to 85 mg/m^3^ at 2.75 m from the source. The low-velocity test for coal resulted in much lower concentrations. Limestone high-velocity results showed that maximum respirable dust concentrations ranged from 11 to 25 mg/m^3^ at 1 m from the source, while the maximum concentrations ranged from 12 to 20 mg/m^3^ at 2.75 m from the source.

It should be noted that there is an anomaly that occurs at location 3 for both coal and limestone low velocity tests. These maximum concentrations are approximately 90 mg/ m^3^ for the coal and 70 mg/m^3^ for limestone, which is much greater than all the other maximum concentrations encountered during their respective tests. Explanations for this phenomenon are not obvious, except that perhaps more dust is generated at the sudden stoppage of the shield advance. This phenomenon is not noticeable for the high-velocity coal and limestone.

These results demonstrate that the shield dust simulator is able to produce consistent results over the shield advance sequence. This capability is critical in order to properly classify the reduction that could occur with various foam applications. The test apparatus automation through computer controls allowed for a consistent and repeated simulated shield cycle.

## Figures and Tables

**Table 1:** Arrestal model AR55 bag house specifications

Model	Airflow (m^3^/s)	External static pressure (Pa)	Motor power (kW)
AR-55	1.2–2.8	1991–3730	11.2

**Table 2:** Vertical liner actuator specifications

Motion	Vertical linear	Horizontal linear
Air actuator type	Air cylinder	Linear motion barrel
Linear air cylinder type	Tie rod	Grooved
Tie rod construction	External tie rods	NA
Bore size	7.62 cm	3.81 cm
Width	8.57 cm	NA
Carriage	NA	21.5 cm
Stroke	21.6 cm	91.44 cm
Retracted	36.96 cm	NA
Extended	58.5 cm	NA
Overall	NA	121.5 cm
Overall height	NA	9.75 cm
Force @ 50 psi	160 kg	44 kg
Force @ 100 psi	321 kg	88 kg
Actuation mechanism	Air extend, air retract	Air extend, air retract
Sensor ready	NA	Sensor ready
Air cushion type	NA	Adjustable cushion
Body material	Aluminum	Aluminum
Carriage material	NA	Aluminum
Guide rail material	NA	UHMW
Tie rod material	Steel	NA
Material	Steel	NA
Diameter	1.9 cm	NA
End type	Threaded	NA
Thread size	1.9–25.4 cm	NA
Air inlet Thread size	1.6 cm	0.64 cm
Thread type	NPT	NPT
Gender	Female	Female
Bearing material	Aluminum	NA
Lateral force capacity	NA	160 kg
Vertical force capacity	NA	160 kg
Load force (cylinder twisting forward)	NA	11.2 kPa

**Table 3:** Keystone Black 325BA and bagged limestone particle size data (cumulative passing)

Particle size diameter (μm)	Bagged coal cum. % passing	Bagged limestone cum. % passing
300.0	100.0	100.0
250.0	100.0	100.0
200.0	100.0	100.0
150.0	100.0	98.7
100.0	100.0	93.8
75.0	100.0	87.9
50.0	99.9	77.6
40.0	99.9	71.7
20.0	82.8	58.3
10.0	46.2	44.5
5.0	24.1	26.8
4.0	19.3	21.7
0.5	0.2	5.2
0.1	0.0	0.4

**Table 4:** Horizontal and vertical actuator position throughout test sequence

Time (s)	Horizontal actuator position (%)	Vertical actuator position (%)
0	0	100
3	0	75
7	60	100
12.25	100	100
12.75	100	75
13.31	50	75
19	0	75
27.5	0	0

**Table 5:** Cycles for each test sequence

Dust type	High-velocity shield cycles	Low-velocity shield cycles
Dust-coal	40	40
Dust-limestone	40	40
